# 

**Published:** 1970-07

**Authors:** 


### CONTENTS

Human Liver Transplantation ... ... ... ... ... ... 63

M. G. Johnson

Government Medical Officer in Lesotho ... ... ... ... ... 69

W. E. Benney

Treatment of advanced Breast Carcinoma with Drostanolone Propionate ... ... ... ... ... ... ... ... ... 75
N. M. Heney

"Reviewing our Series, Three Cases only died" ... ... ... ... 77

Alan B. Raper

Scientific Measurements in Sociology ... ... ... ... ... 81

John Clutton-Brock

Obituary Notices ... ... ... ... ... ... ... ... 82

Wisdom from the Past ... ... ... ... ... ... ... 84

The Medical Library ... ... ... ... ... ... ... ... 86

Society Reports ... ... ... ... ... ... ... ... 87

Notes and News ... ... ... ... ... ... ... ... 88

F. Bailey & Son Ltd., Dursley, Glos.

